# The importance of extended working hours for work-related injuries

**DOI:** 10.5271/sjweh.3981

**Published:** 2021-08-31

**Affiliations:** Anne Helene Garde, PhD, The National Research Centre for the Working Environment, Copenhagen, Denmark. Email: ahg@nfa.dk

Worldwide, there are an estimated 374 million non-fatal work-related injuries with >4 days of absences from work (1) and more than 3000 fatal work injuries in European countries each year (2). The human cost of this is vast, and the economic burden of poor occupational safety and health practices is estimated to be about 4% of global gross domestic product (GDP) each year (1). There is a need to identify risk factors of work-related injuries as such knowledge may help decrease the number of injuries.

A promising candidate for such a risk factor is extended working hours, which leave less time outside work and thereby less time for sleep and restitution. The resulting sleepiness can in turn cause poorer cognitive performance and thus constitutes a plausible mechanism linking long working hours to accidents. Being awake for 18 hours has been shown to impair performance corresponding to a blood alcohol content of 0.05 % (3), and long daily working hours (12-hour shifts) have been associated with impairment of alertness and performance (4). This line of effects may link extended working hours to acute safety risks as well as possibly long-term health effects. In addition, reduced performance not only puts the person who works the long hours at risk, it could also have implications for the safety and health of others. Extended working hours could, for example, affect patient and traffic safety (5). In a study of nurses working 12-hour night shifts, almost all reported having at least one motor vehicle accident or near accident during the previous 12 months driving to or from work (6).

Undoubtedly, there is an upper limit to how long it is possible to work as this is limited by the number of hours in the day and the need for recovery, and working hours has for long been a focus of regulation. Indeed regulation of working hours was part of the 1802 Health and Morals of Apprentices Act, which was the very first piece of factory legislation in the United Kingdom. It prevented apprentices in cotton mills from working at night and for >12 hours a day (7). Today, the Working Time Directive (2003/88/EC) regulates the duration of working hours in Europe (8).

In this issue of *Scandinavian Journal of Work, Environment and Health*, Matre and colleagues (9) report the results of a highly welcomed systematic review and meta-analysis on extended working hours and safety incidents (accidents, near-accidents, safety incidents and injuries). They reviewed studies on extended working hours in terms of both daily and weekly working hours and found that both long daily (>12 hours) and weekly (>55 hours) working hours are associated with increased risk of safety incidents (9). The review adds to several other recent systematic reviews regarding extended weekly working hours performed by the World Health Organization, the International Labor Organization and the IPD-Work consortium, which have examined the associations between long working hours and primarily long-term health outcomes such as cardiovascular disease (10–14), cancer (15), type 2 diabetes (16), alcohol consumption (17) and depression (18, 19).

From an epidemiological perspective, the analysis of extended working hours has advantages compared to analysis of many other work-related exposures. Working hours are relatively easy to measure. All employees can measure an hour objectively and with high precision. Duration of working hours can also be assessed with relatively high confidence by answering a single question. This makes the assessment of extended working hours attractive in general population surveys resulting in large cohorts with a standardized measure across jobs, gender, age, socioeconomic class and other contextual factors such as regional cultures.

However, there are several methodological challenges that need to be solved in order to move the field forward. Importantly, as also pointed out by Matre and colleagues, extended work hours is highly intertwined with other measures of working hours such as short time between work shifts (quick returns) and working outside ordinary day work, eg, during the evening or night, which in itself has been associated with increased risks for health and safety. In addition, long work days do not necessarily imply long weekly working hours. The same weekly working hours can be achieved by having longer but fewer work days (compressed work weeks). Such compressed work weeks are advantageous in several ways. The lower number of work days gives employees more days without work. It decreases total commuting time – and the risks and environmental exposures associated thereof – and has been suggested as a means of reducing traffic congestion, although the effects are debated (20). Compressed work weeks are a well-established type of work schedule, eg, in the US Federal Government (21). It is therefore important to be able to disentangle the differences in risks by studying specific combinations of working hours.

Another challenge is to determine whether the extended working hours are the actual cause of health safety outcomes. While there is a plausible link from extended working hours to accidents through lack of recovery and reduced performance (4), there may also be alternative mechanisms, in particular when it comes to more long-term effects. Extended weekly working hours, especially when related to overtime work, may be due to high workload. High workload may elicit feelings of being stressed at work, for example in the form of job strain that has been associated with increased risk of cardiovascular disease (22). In addition, long working hours imply longer exposure time to all other work environmental risk factors, such as chemical and physical exposures.

Even if working time can be measured in a standardized way across different cultures and traditions, there are regional differences in what is considered the normal duration of working hours. For example, the proportion who worked >55 hours per week in 2016 was about 12% in South-East Asia and 3.5% in Europe (14). Furthermore, the reasons for extended working hours, may differ between countries and regions, and this may in turn modify the relationship between extended working hours and health. In countries with high social security and minimum wage, extended work hours is likely to be a more voluntary choice compared to countries or jobs where workers have extended working hours due to financial necessity.

When it comes to measurement of working hours, Matre and colleagues (9) point to payroll-data as the golden standard. This is particularly true when the registration is linked to payment of wages, eg, in terms of overtime work. However, the validity of length of daily and weekly working hours is expected to be lower in jobs where unpaid extra working hours are more common (eg, among physicians and other academic personnel) and if hours from secondary jobs are not included (23). Even so, cohorts based on day-to-day registrations from payroll data have in recent years moved the field considerably forward due to highly detailed information on working hours for every day. The advantages for studies of associations between working hours and accidents include better information on temporality and decreased risk of misclassification. Thus, recent studies have analyzed associations of working hours the previous week(s) and risk of accidents on a given day (24, 25). The very detailed data are particularly important when analyzing the effect of extended working hours on accidents, since the effect must be assumed to be short term. Day-to-day registrations of payroll data also open the door to using more advanced designs, eg, case–crossover design (24, 26) that eliminates confounding by time-invariant variables. Finally, day-to-day payroll data allow detailed adjustment for other working hour characteristics and give the possibility to analyze duration of daily working hours in more detail. This will lead to more precise analyses on the association between working hours and the risk of work-related injuries and the potential for reducing the risk in the future.

The author thanks Marie Aarrebo Jensen, Ann Dyreborg Larsen, and Helena Breth Nielsen for their thoughtful comments on the initial draft.
